# Seismic low-velocity equatorial torus in the Earth’s outer core: Evidence from the late–coda correlation wavefield

**DOI:** 10.1126/sciadv.adn5562

**Published:** 2024-08-30

**Authors:** Xiaolong Ma, Hrvoje Tkalčić

**Affiliations:** ^1^ Research School of Earth Sciences, The Australian National University, Canberra 2601, ACT, Australia.; ^2^ Southern Marine Science and Engineering Guangdong Laboratory (Guangzhou), Guangzhou 511458, China.

## Abstract

Thermochemical inhomogeneities in the Earth’s outer core that enhance our understanding of the geodynamo have been elusive. Seismic constraints on such inhomogeneities would provide clues on the amount and distribution of light elements in the core apart from iron and nickel. Here, we present evidence for a low-velocity volume within the outer core via the global coda correlation wavefield. Several key correlogram features with a unique sensitivity to the liquid core show variations with wave paths remarkably slower in the equatorial than polar planes. We constrain a torus structure at low latitudes with ~2% lower velocity than the surrounding liquid outer core via waveform modeling. We propose a thermochemical origin for such a low-velocity torus, providing important constraints on the dynamical processes of the Earth’s outer core.

## Teaser

The lateral inhomogeneity in the Earth’s outer core is characterized using the earthquake coda correlation wavefield.

## INTRODUCTION

The generation and maintenance of the Earth’s magnetic field are attributed to the vigorous fluid convection in the outer core (OC) via the geodynamo mechanism. Such fluid convection can be driven by thermal and compositional buoyancy sources (*1*–*3*). The Earth’s liquid iron-alloy OC was previously considered well-mixed and homogeneous due to its vigorous convection (*4*). However, it is still debated whether volumetric inhomogeneities could exist (*5*–*13*). Some studies have proposed and demonstrated several plausible inhomogeneity origins, including a tangent cylinder structure due to an inviscid convective fluid under the Earth’s rotation and the presence of a solid inner core (IC), gravitational perturbations induced by the asphericity of the mantle (*14*), heat flux variations through the core-mantle boundary (CMB) (*15*), and localized concentration of light elements released by the IC (*16*). Placing seismic constraints on the OC inhomogeneities could enhance our understanding of the Earth’s geodynamo mechanism, core dynamics, and core-mantle interactions. Therefore, it is vital to conceive observational probes to comprehend the distribution, magnitude, and morphology of such inhomogeneities.

We devise an approach based on recent advances in the Earth’s coda correlation wavefield to illuminate the OC structure. This mathematical coda correlation wavefield, constructed by cross-correlating hours of long-lasting earthquake late-coda waves using a large number of seismic stations, complements the seismic wavefield and can be represented by a global correlogram (*17*–*19*). The global correlogram is a two-dimensional (2D) graphical expression of coda cross-correlation stacks as a function of inter-receiver distance. Prominent signals, named correlation features, can be observed in the global correlogram and manifest similarity with seismic phases in the regular seismic wavefield. A given coda correlation feature is formed due to interactions of many cross-terms of multiply reverberating body waves through the Earth with a common ray parameter (*20*–*22*). In this study, we use time variations between the correlation features, sampling different parts of the OC in a way the direct seismic phases cannot due to the uneven distribution of earthquakes and seismic stations worldwide. We then use 3D waveform modeling to constrain ~2% strength of lateral heterogeneity within the OC. We further demonstrate that the crust, mantle, and IC influence on our data are minimal. The preferred OC model has a low-velocity torus-shaped structure beneath the CMB at low latitudes. The remaining part displays weak high-velocity variations relative to our reference model.

## RESULTS AND DISCUSSION

### Time variations of correlation features in global correlograms

**Fig. 1.**
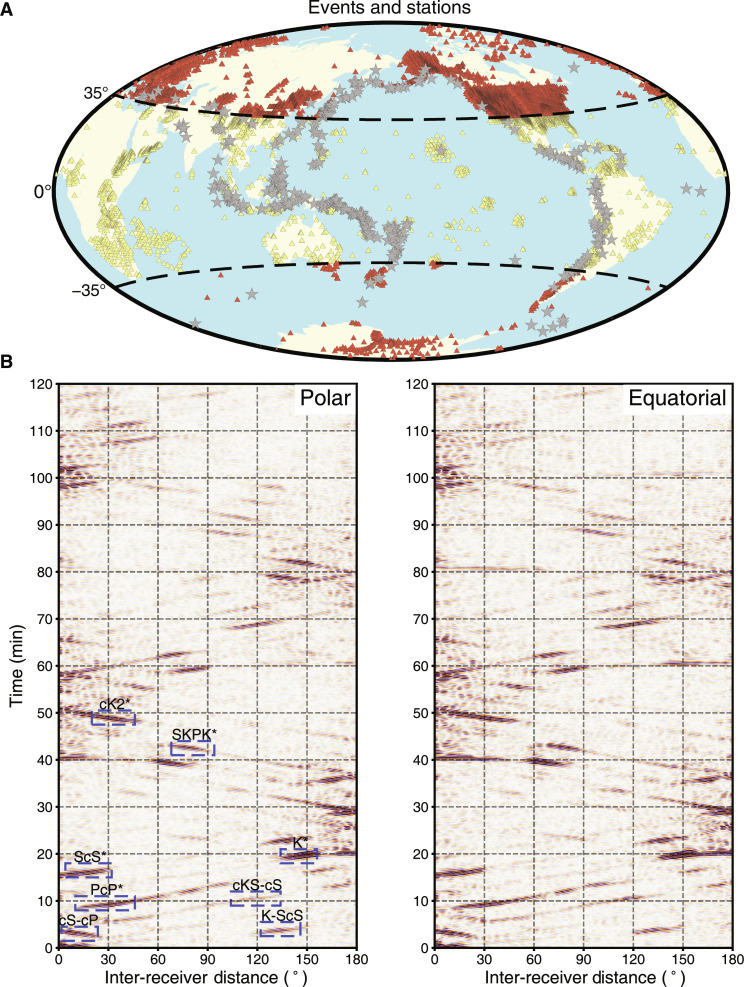
Global stations, event distribution, and observed global correlograms for “polar” and “equatorial” crossings in the OC. (**A**) A geographic map of the locations of receivers and earthquakes (red stars) and stations (triangles) used in this study. The stations are divided into two groups (coral and khaki) based on their latitudes (see the main text for definitions). (**B**) The stacked global correlograms between 0 and 7200 s after the correlation origin time as a function of inter-receiver distance from 0° to 180°. The blue dashed rectangles denote the targeted correlation features (PcP*, ScS*, cS-cP, K*, SKPK*, cK2*, cKS-cS, and K-ScS) in this study. The featured correlograms are constructed using polar (left) and equatorial (right) crossings in the OC (see the main text for the definitions).

We compute the coda correlation wavefield and obtain the corresponding correlogram using a global dataset at periods between 15 and 50 s. We divide the worldwide stations into two groups based on their latitudes. Stations with latitudes between −35° and 35° are empirically classified as the “equatorial group,” while stations with latitudes higher than 35° are the “polar group” (Fig. 1A). Subsequently, we compute the global correlograms for each group (Fig. 1B) following the procedures in Materials and Methods and compare several coda correlation features in the correlogram between the two groups [see (*19*, *20*, *23*) for the naming convention]. Our observations in this study are the travel time differences of the correlation features (measured by the waveform shift) between the polar and equatorial groups.

**Fig. 2.**
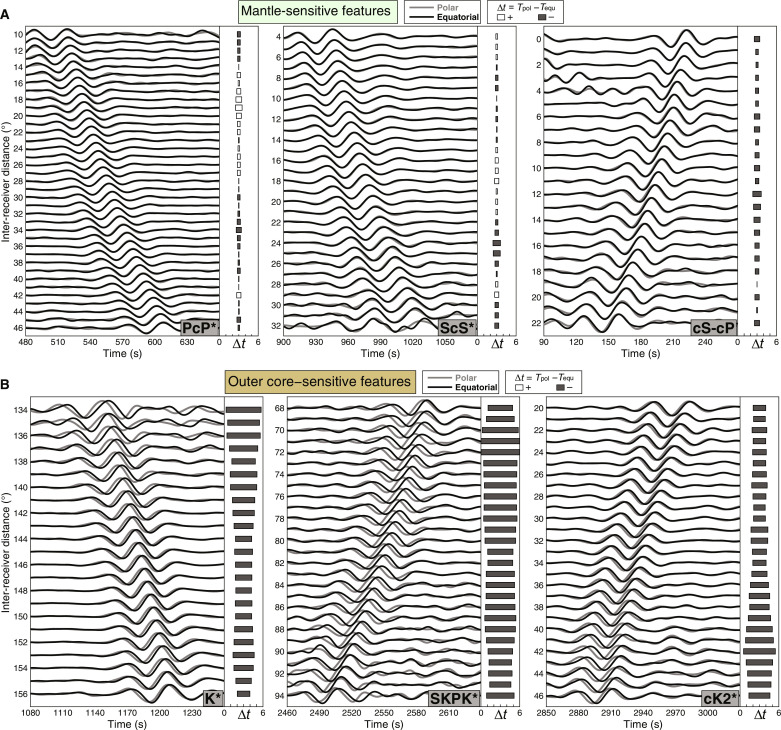
Observed mantle-sensitive and OC-sensitive correlation features. (**A**) Waveform comparisons of selected correlation features PcP*, ScS*, and cS-cP from polar (gray) and equatorial (black) groups. These features are mainly sensitive to the mantle structure. The horizontal bars in each side panel indicate the calculated travel time differences of the correlogram features between the polar and equatorial groups [gray bars are negative (polar faster than equatorial), and white bars are positive time variations]. (**B**) Similar to (A), waveform comparisons for K*, SKPK*, and cK2*, the features exposed to the OC structure.

PcP*, ScS*, and cS-cP features (sensitive mainly to the mantle structure) display almost identical travel times between the two groups (Fig. 2A). In contrast, some coda correlation features, with substantial sensitivities in the OC (e.g., K*, SKPK*, cK2*, cKS-cS, and K-ScS) differ between the two groups: The polar group is notably faster than the equatorial group by an average of 3 to 5 s (Fig. 2B). Such robust time differences of OC-sensitive features are verified by testing other geographical divisions of stations (fig. S1) and randomly selecting a subset of earthquakes (fig. S2).

Although similar to the body-wave travel time sections, these features represent the similarity (coherence) between the body waves, fundamentally different from the regular seismic phases. Considering the complex propagation paths of these features’ constituents (fig. S3), we focus on K* for further analysis because this feature is more prominent in the global correlogram than others. Note that we use the name K* to represent the feature in this distance range and time window hereafter, although other features exist in the corresponding distance range (e.g., cPc2-cKS at shorter distances).

### Waveform modeling through different models

**Fig. 3.**
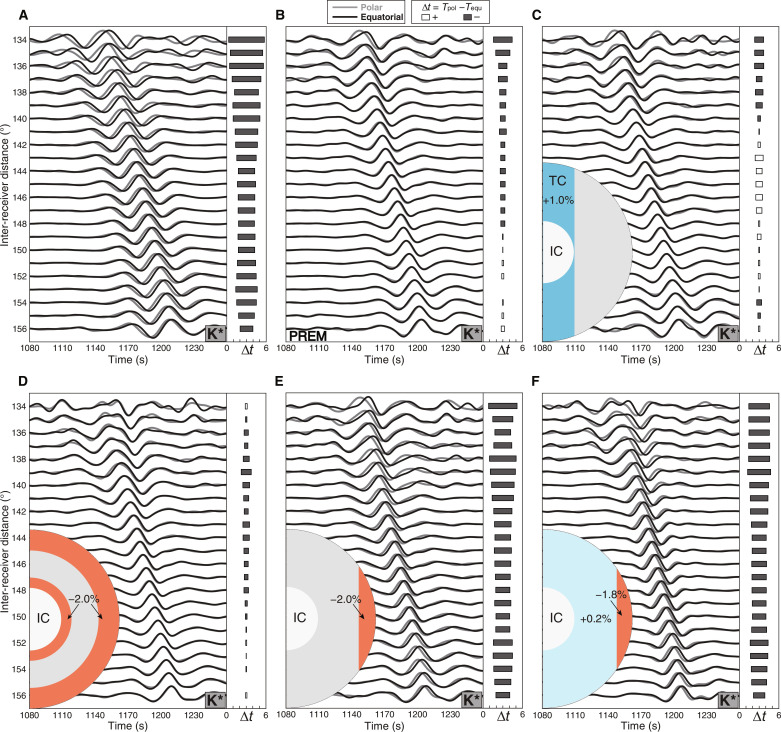
Comparisons of the polar and equatorial groups’ waveforms for observed and synthesized K* feature for different models. The gray and black lines represent the polar and equatorial groups, respectively. The horizontal bars in each side panel indicate the calculated travel time difference of the features between the polar and equatorial groups (see Fig. 2 caption). (**A**) The observed K* correlation feature. (**B**) PREM (Preliminary Reference Earth Model) with Earth’s ellipticity. (**C**) A tangent cylinder model synthetics for the 1% elevated seismic velocity within the cylinder relative to the background model PREM. The light blue area indicates the OC volume with positive seismic velocity perturbations. (**D**) The orange area indicates the model with low seismic velocities (−2% relative to PREM) at the top and bottom of the OC. (**E**) The model with a low seismic velocity of −2% within the equatorial torus using PREM as a background model. (**F**) The model with a low seismic velocity of −1.8% within the equatorial torus and a weakly high velocity of +0.2% in the remaining part in the OC using CCREM (Coda Correlation Reference Earth Model) as a background model.

We first demonstrate that 1D model of Earth cannot explain our observations (Fig. 3B). Then, we scrutinize multiple causes that could explain the time variations for these features. They are (i) Earth’s ellipticity, (ii) mantle heterogeneity, (iii) the CMB topography, (iv) OC inhomogeneities, and (v) IC structure. We test them based on a forward modeling approach and calculate the L2-norm misfit between the synthetic and observed data points of the correlation feature travel time variations (see figs. S7, S12, S14, and S16 and tables S1 and S2). We synthesize the long-duration coda waves, taking into account the Earth’s ellipticity and several global tomographic models in the 3D simulations (fig. S6). The same processing procedures are applied to the synthetic and the observed waveforms. We find no pronounced time shifts for the synthetic K* feature (fig. S6), meaning that the Earth’s ellipticity and mantle heterogeneity could not cause the time variations observed between the two groups. We conclude that the above two effects are laterally averaged out by stacking millions of cross-correlations from many worldwide stations in diverse directions.

The CMB topography effect on the correlation features is also modeled via synthetic simulations. Only a substantial elevation of CMB (>20 km) can account for the time variations of K* (fig. S9A). However, this CMB topography also causes the time shifts of PcP* between the two groups, which are not observed in the data (fig. S9B). Besides, the correlation feature PcP*, sensitive to the mantle structure, CMB topography, and Earth’s ellipticity show no pronounced time variations. This further proves that the effects of these three factors are minimal, and the origin of our observations must be sought in the Earth’s core. Subsequently, we demonstrate that these selected features are not sensitive to the IC structure (fig. S10). Thus, we can exclude the effects of the IC anisotropy and heterogeneities (*24*, *25*) on the time variations between the polar and equatorial groups. In summary, the Earth’s ellipticity, mantle heterogeneity, CMB topography, and IC structure cannot reconcile the observed difference in K* between the two groups.

**Fig. 4.**
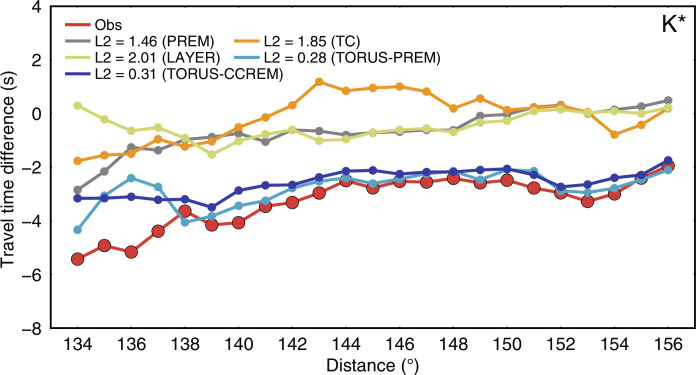
Travel time variations for the K* feature between the polar and equatorial groups for different models relative to the observations. TC represents the tangent cylinder model. LAYER indicates the layered model with low seismic velocities at the top and bottom of the OC. TORUS-PREM and TORUS-CCREM denote the models with low seismic velocities within the equatorial torus using PREM and CCREM as background models, respectively.

**Fig. 5.**
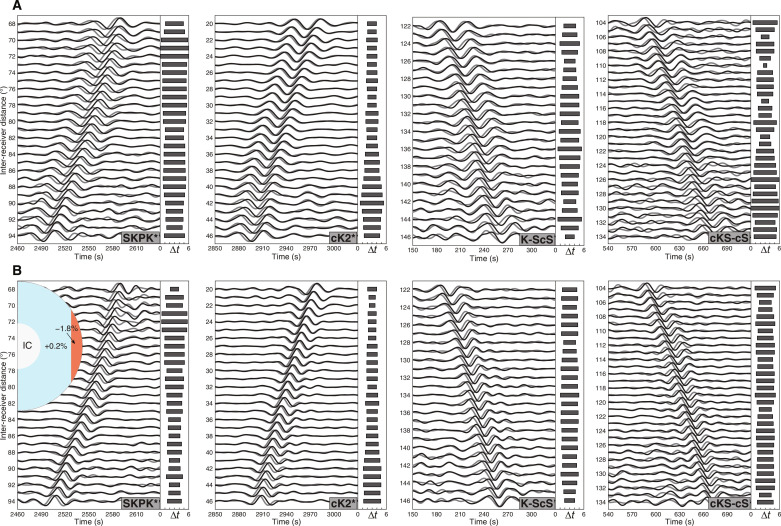
Observations of selected correlation features and synthetic waveforms for the preferred OC model. (**A**) Observed waveforms of four correlation features: SKPK*, cK2*, K-ScS, and cKS-cS, for the polar (gray) and equatorial (black) groups. (**B**) The preferred OC model and the corresponding synthetic waveforms for the four selected features. Here, we use CCREM as a background model in the simulation to fit both the time variations and absolute travel times of the correlation features better simultaneously. In the OC model, we add a 150-km-thick zone with the velocity perturbations increasing from −1.8 to +0.2%.

**Fig. 6.**
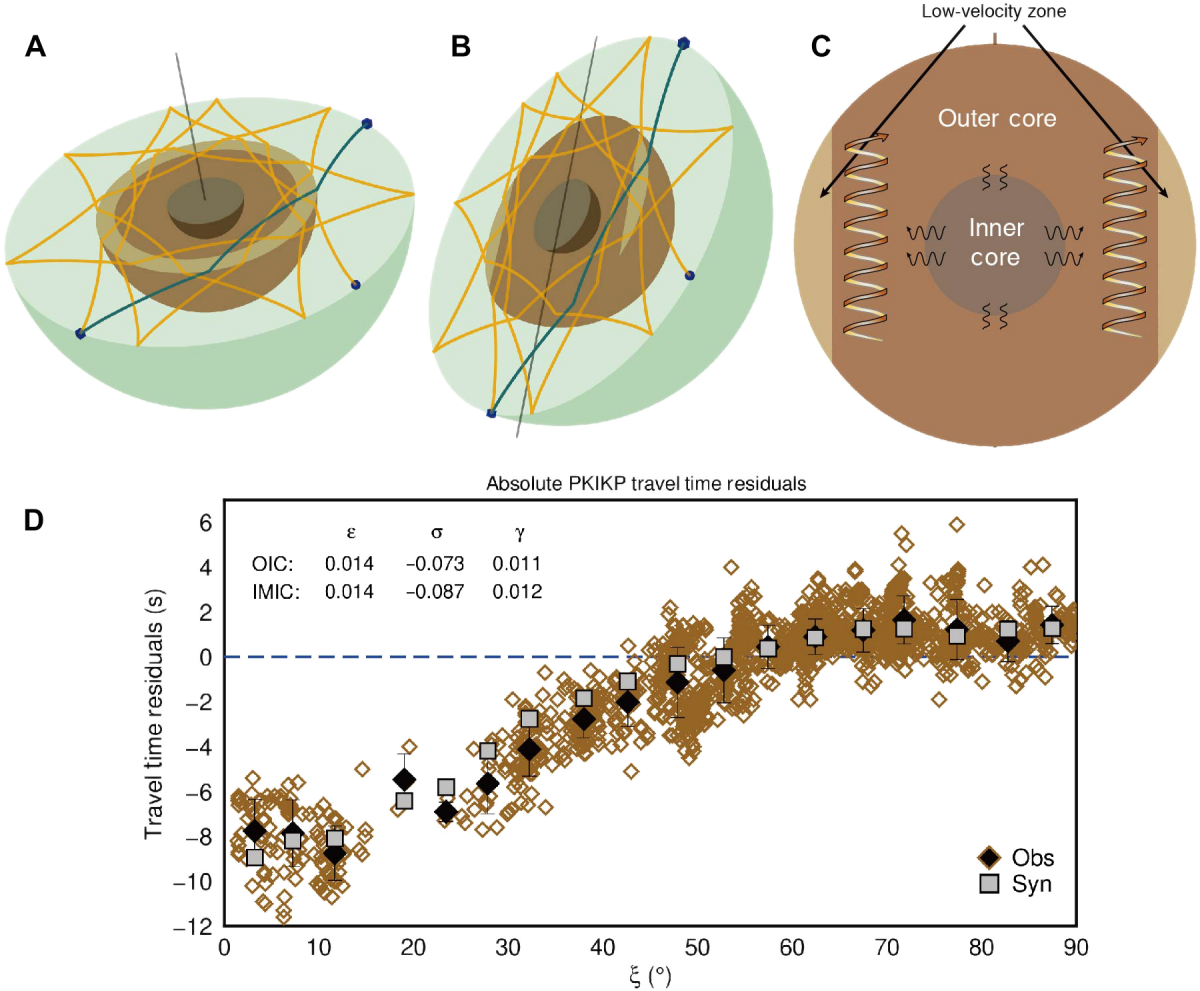
Ray path of K* feature, schematic of the OC heterogeneity, and absolute PKIKP travel time residuals. A simplified schematic illustration of the generation of coda correlation feature K* from the constituent (PKP)_10_-(PKP)_9_ for the two groups: equatorial (**A**) and polar (**B**). The light brown–shaded areas denote the low-velocity torus in the OC. The blue cubes are the seismic stations, and the ball is the event. The dark solid line is the Earth’s rotation axis. (**C**) Cross section of the OC with the inferred low-velocity torus (light brown region) beneath the equatorial CMB. Helical curves represent the convective flow in the OC [see (*30*, *34*, *35*, *51*)]. Wiggly lines at the IC boundary represent higher heat transfer in equatorial than polar regions. (**D**) Observed and predicted absolute PKIKP travel time anomalies as a function of the angle between the PKIKP ray path in the IC and the Earth’s rotation axis. Brown diamonds show the observations (*28*). Black diamonds are binned data with 1-σ error bars and a bin size of 5°. The gray squares indicate the synthetic binned data calculated for the model, including mantle heterogeneity, OC torus, and IC anisotropic structure using the same event-station configurations as the observations. The anisotropic parameters of the IC model are shown on the top left.

After excluding possible causes for the time variations in the correlation features, we now focus on volumetric inhomogeneities in the OC (Figs. 3, C to F, 4, and 5). First, we test several possible heterogeneous structures in the OC, including the tangent cylinder, outermost-core stratification, polar cap, and columnar heterogeneity models. None of these models can explain the observed time variations of K* (Fig. 3, C and D, and figs. S11 and S12). In particular, the widely proposed OC tangent cylinder structure in geodynamic simulations does not generate the observed variations in the K* feature. We then propose a simple OC structure based on recent geodynamical studies that can fit well the observed time differences in the selected correlation features using the Preliminary Reference Earth Model (PREM) (*26*) as a reference model (Fig. 3E and fig. S13). This model displays an equatorial torus of about 2% negative velocity perturbation, with the thickest part reaching ~600 km beneath the CMB in the OC. In this model, the K* features in the equatorial group can sample the low-velocity torus beneath the CMB at the low latitudes well compared to K* in the polar group with a much poorer sampling of that volume. We show only one configuration of wave paths in the OC for K* in Fig. 6 (A and B). However, understanding the full sensitivity of K* to the OC structure requires a more detailed analysis of cross-terms of body waves contributing to the K*.

PREM cannot fit the absolute travel times of the correlation features well due to its relatively fast velocity profile. Thus, we use the CCREM (Coda Correlation Reference Earth Model) (*27*) as our background model to improve the fit further. The CCREM is constructed to fit optimally as many correlation features as possible in the global correlogram. We then explore more models by varying the thickness, velocity variation, and shape of the OC model (fig. S14). We find that only the torus model can explain the observations satisfactorily. Therefore, we test more variants of the torus model by changing the thickness and velocity perturbation (fig. S15). Although some models show a similar fit to the observations (fig. S16 and table S2), we can exclude the models that cannot match the absolute travel times of some features (fig. S17). Note that it is challenging to determine the best-fitting model due to the trade-offs in the lateral heterogeneity’s velocity perturbations, shape, and volume. After testing the end-member models from the tangent cylinder to the torus model, we chose a representative conceptual model that explains our observations well (Figs. 3F and 5). The preferred model consists of an equatorial torus-shaped low-velocity zone of −1.8% restricted to low latitudes and a high-velocity region elsewhere in the OC with a weak velocity variation of 0.2%. The schematic of the OC heterogeneity is shown in Fig. 6C.

The low-velocity volume in the OC makes a difference in inferring the IC anisotropic structure from the phase PKP travel time dataset, typically assuming a homogeneous OC. Therefore, we further compare the synthetic data calculated for the preferred OC model with the observed absolute PKIKP and differential PKPab-PKIKP travel time residuals (*28*, *29*). We find that the torus model could reasonably predict the traditional travel time observations when a cylindrically anisotropic IC is included (Fig. 6D and figs. S18 and S19). Although the synthetic data cannot perfectly match the observations, this could be due to uncertainties in the existing mantle models and travel time data errors. Besides, the torus-shaped OC model is proposed to explain the time variations in the coda correlation features with periods of 15 to 50 s, which have different sensitivities to Earth’s structures compared to much shorter period body waves. We expect the OC model’s fine details to be constrained when the trade-offs in the lateral heterogeneity’s velocity perturbation, shape, and volume are resolved in future studies.

### Implications for OC dynamics

Numerical simulations have shown that a stable dual structure, which contains inner radial plumes aligned with the Earth’s rotation axis and an outer cylindrical zonal flow, can exist in the OC (*30*). Such a dual structure is comparable to our model’s equatorial torus-shaped low-velocity zone and columnar weakly high-velocity region (Fig. 6C). However, the lateral heterogeneity in the OC cannot be caused by thermal convection alone because the temperature is demonstrated to have only a marginal impact on the P-wave velocity of liquid iron (*31*). Light elements from the IC crystallization or mantle-core reactions are expected to affect the seismic velocity in the core more prominently. We thus prefer the thermochemical origin for lateral heterogeneity in the OC.

Latitude-dependent flow regions have been demonstrated to exist in the convective OC (*32*, *33*). Such a latitudinal dependence could present differing convection dynamics and result in the nonuniform transportation of light elements. Along with these processes, a higher concentration of light elements beneath the CMB would be expected in the equatorial direction due to the greater heat transfer in the equatorial than in the polar regions (*34*, *35*). Thus, the accumulated light elements could cause an average compositional difference between low-latitude regions and the rest of the OC, which would likely result in low-velocity zones observed and modeled here. Moreover, the light element exchange from the mantle-core reaction can contribute to the formation of low-velocity zones (*36*). It should be noted that the resultant seismic wave speed depends on the core’s bulk composition and the impact of different light elements on the bulk modulus and density of liquid iron alloy (*36*–*39*). If the sound velocity and density scale according to Birch’s law, then this would imply lateral density variations several orders of magnitude larger than is permitted by the dynamics (*4*). However, we cannot constrain the density variations in the OC because the coda correlation features have less sensitivity to the density perturbation than the velocity perturbation.

Seismological studies have reported different low-velocity profiles in the outermost core relative to PREM (*40*–*44*). Although lower seismic wave speeds could be associated with a stratified layer at the core’s top (*45*–*50*), such a 1D stratification model cannot explain our observations (Fig. 3D and fig. S14C). Alternatively, the stratified layer near the top of the core could be expected at low latitudes, possibly due to the penetration of thermal winds into the stratified region at high latitudes (*51*). Moreover, regional stratification at the top of Earth’s core beneath the Pacific Ocean has been suggested because of CMB heat flux variations (*15*). Nevertheless, we do not observe notable longitude-dependent K* feature variations by further dividing the stations longitudinally (fig. S1), suggesting no or weak lateral variations within the torus-shaped volume. Nevertheless, we cannot completely exclude this possibility if the regional stratification is thin compared with the wavelength of our data.

To summarize, we have revealed a latitudinal pattern of inhomogeneity in the OC using the coda correlation wavefield. Such heterogeneity was mentioned in several previous studies (*52*, *53*). However, more seismological investigations are required to confirm the existence of such a structure in the OC, considering the limited coverage of body-wave phases and effects from strong heterogeneities in the lowermost mantle. We anticipate that our findings can serve as the starting point for future investigations of lateral heterogeneity in the evolution of OC dynamics and composition. High-pressure experiments and extensive geodynamic simulations would be essential for future studies to consolidate our inference of OC inhomogeneity configuration from the seismic coda correlation wavefield. The density and velocity variations in the OC need to be simultaneously constrained by considering normal mode, body wave, and coda correlation data to bring us a step closer to reconciling the seismological observations with geodynamical constraints. A feasible physical mechanism is required to explain such a structure combining the abovementioned disciplines.

## MATERIALS AND METHODS

### Construction of global correlogram

We obtained the vertical component seismograms recorded at global seismic stations from large earthquakes with moment magnitude ≥ 6.8 between 2000 and 2021. First, we corrected the instrument response and removed the mean and linear trend of the raw seismograms. Then, we selected the waveforms in the time window of 3 to 9 hours after the event origin time. This portion of the seismogram is usually regarded as the earthquake’s late coda. The waveform data were processed following the procedures in previous studies (*20*, *54*). We applied the temporal normalization and spectral whitening methods to seismograms of all receivers and calculated cross-correlations by multiplying the whitened spectrum of one receiver with the complex conjugate spectrum of another. Then, the spectral cross-correlations were inversely Fourier-transformed and folded at the time zero to yield cross-correlation functions in the time domain. Subsequently, the cross-correlation functions for receiver pairs were linearly stacked in inter-receiver distance bins with an interval of 1° and band-pass filtered in the period band of 15 to 50 s for a single-event correlogram. Because the late coda in earthquake waveforms consists mainly of strong reverberating energy confined in the great circle plane (*55*), we computed the cross-correlation functions only when the event is proximal to the great circle plane defined by the receiver pair. Empirically, we choose the receiver pair if the spherical distance from the event to the great circle path is less than 5°. Then, the individual global correlograms are stacked over multiple events. The stacked global correlogram displays prominent correlation features similar to previous studies (*20*, *23*). All these features are formed due to the interaction of many pairs of phases with the same slowness (*18*, *20*, *56*), which are not “reconstructed body waves” under the principle of Green’s functions (*19*).

The early-emerging features K*, SKPK*, cK2*, K-ScS, and cKS-cS, which are OC-sensitive features, are selected as our observations to demonstrate the possibility of lateral heterogeneities in the fluid OC because they are prominent in the global correlogram. We excluded prominent correlation features such as I* and I2* (*57*) since they can sample both the complex anisotropic IC and OC.

### Grouping the global stations

First, we tested whether the OC has a longitude-dependent velocity structure by longitudinally dividing the stations into three individual groups with a range of 120°. The longitudinal ranges for these three groups are 40° to 160°E, 160° to 280°E, and 280° to 40°E, respectively (fig. S1A). Then, the global correlograms are generated for the three groups following the above procedures. We do not observe notable time variations in K* features between these three groups (fig. S1B), which excludes the possibility of longitude-dependent velocity structures in the OC. We then divided the global stations into two groups based on their latitudes to test whether the OC has latitude-dependent heterogeneities. The stations are classified as the polar and equatorial groups based on their latitudes. In this case, we can observe clear time variations between the core-sensitive correlation features in the two groups. We further group the polar station pairs into two subgroups, named AK (connecting the stations in the Antarctic to the stations in Alaska, longitude: 190° to 240°E) and EU (connecting the stations in the Antarctic to stations in Europe, longitude: 0 to 50°E), respectively. Then, we compute the coda correlation for these two polar paths and find that the K* in the AK group exhibits similar travel times to that in the EU group (fig. S4). This indicates that the subducted slab beneath Alaska, which is thought to cause strong anomalies in PKPbc(ab)-PKIKP differential travel times (*58*), does not remarkably affect the K* feature.

### Synthetic simulations

Both 1D and 3D forward modeling approaches are adopted for the synthetic simulations. We used AxiSEM3D (*59*), a hybrid of spectral element and pseudospectral method, to synthesize the observed correlation features by simulating the seismic wavefield in 3D heterogeneous Earth models. Initially, the PREM (*26*) is set up as the 1D background model. Then, we synthesized the coda correlation features by testing different 3D heterogeneous models. The computational mesh sizes are adapted according to the input model for a dominant period of 12 s. In the simulation, the moment tensor solutions are used based on the Global Centroid Moment Tensor catalog (*60*), and seismic receivers are located at their actual geographic coordinates. Because of the heavy computational burden, we computed 9-hour vertical component synthetic seismograms for only 10 events from the highest-quality event catalog (*23*). The synthetic data were then processed identically to the observations.

Each of these 10 earthquakes is sufficient to generate a high-quality correlogram equal in quality to that constructed from many events (*23*). Similarly, the observed correlation feature K* from these 10 events displays the time variations between the polar and equatorial groups as observed when using many events (fig. S5A). In addition, we show the K* waveforms of each event from the two groups (fig. S5, B and C). Time variations of K* among 10 events can be observed, which we would expect to be due to mantle heterogeneity and different event-station configurations. However, the stacking processes over multiple events minimize such effects on the correlation features.

We calculated the L2-norm misfit for data points of the travel time variations of the correlation feature between the polar and equatorial group to quantitatively measure the fit for the model as in the following

$${\text{Fit}}_{L2}=\frac{\text{Norm}\left(\text{Syn}-\text{Obs}\right)}{\sqrt{\text{Norm}\left(\text{Syn}\right)\;\text{Norm}\left(\text{Obs}\right)}}$$

where Syn and Obs indicate the synthetic and observed travel time variations of the correlation feature between the polar and equatorial groups.

We calculated the synthetic correlation feature K* for the polar and equatorial groups through PREM, taking into account the Earth’s ellipticity. In the simulation, we used the WGS84 (World Geodetic System 1984) coordinate system for the ellipticity correction, meaning that the Earth’s ellipticity is approximately 0.00335. Next, to test the mantle heterogeneity effect on the correlation features, we computed the synthetic waveforms for three tomographic models, LLNL-G3Dv3 (*61*), TX2019slab (*62*), and SP12RTS (*63*). Furthermore, we performed the simulation for a modified TX2019slab model by multiplying the velocity perturbations by a factor of 5 to exaggerate the mantle heterogeneity magnitude. However, all the above tomographic models cannot explain our observations well (fig. S6). Moreover, we test a model with two low-velocity (−2%) cuboids with thicknesses of 1500 and 500 km, respectively (fig. S6F). The two low-velocity cuboids roughly represent the P-wave equivalent to LLSVPs (large low–shear velocity provinces) at the CMB. No substantial time variations of the K* features are generated between the polar and equatorial groups for this model, either. The plot of the L2-norm fit for the K* feature through the abovementioned models is shown in fig. S7. Some regional heterogeneity models, including a large-scale 100-km-thick ULVZ (ultralow velocity zone) with a compressional wave velocity perturbation of −10% and shear wave velocity perturbation of −30% beneath Africa and a 200-km-thick slab with a compressional wave velocity perturbation of 6% and shear wave velocity perturbation of = 6% spanning about 2000*3100 km^2^ beneath Alaska, are also tested. We find that these regional models cannot explain the observed time variations due to the global stacking of coda correlations from a large number of stations and events (fig. S8).

We then took into account the CMB topography in the simulation. Taking a conservative approach with a large CMB topography (20 km), noting that a recent study found a root-mean-square CMB topography perturbation of 4.5 km (*64*), can generate the time variations of the K* feature (fig. S9A). However, such a model could also cause substantial time variations in PcP* between the two groups (fig. S9B), which are not observed in the data. Moreover, the effects of regional CMB topography variations (a few kilometer variations on the lateral scale of several hundred kilometers) are averaged out by stacking the coda correlations in the period range from 15 to 50 s from all the global stations.

We also used the Yspec code (*65*) to calculate the 9-hour-long synthetic waveforms for spherically symmetric Earth models with the highest period of 8 s. We demonstrated that K*, SKPK*, cK2*, K-ScS, and cKS-cS features are insensitive to the IC structures by varying the velocity structures in the IC. Then, we generated these synthesized correlation features for both CCREM (*27*) and CCREM-perturbed models. In the two CCREM-perturbed models, the P-wave velocity in the IC is varied by −1.0% and +1.0%, respectively. We find that these features are almost the same for the three IC models (fig. S10), indicating that they are not affected by the IC structure.

### IC anisotropy

We find that an anisotropic IC is required to better fit the observed PKIKP travel time residual data (*28*) if the torus-like heterogeneity exists in the OC. Here, we assume a cylindrical anisotropic structure in the IC. In general, in an axisymmetric anisotropy model, the P-wave velocity perturbation to an isotropic velocity can be expressed as a function of ξ, which is the angle of the ray path of PKIKP with the Earth’s rotation axis as

$$\frac{\delta V_{p}}{V_{p}}=\varepsilon {\text{cos}}^{2}\xi +\sigma {\text{sin}}^{2}\xi \;{\text{cos}}^{2}\xi +\gamma$$

where ε and σ are parameters related to the Love’s parameters (*66*), and γ is a baseline shift.

Because of the large scatter in the observations, we bin the data points using a bin size of 5°. We subsequently compute the synthetic PKIKP data taking into account the tomographic model TX2019slab (*62*), the preferred OC heterogeneity, and an anisotropic IC. We grid search for the optimal parameters of a cylindrically anisotropic IC by fitting the binned PKIKP data for different models. We first determine the anisotropic parameters for a bulk IC. Then, we refine the IC model, including an innermost inner core (IMIC) with a radius of 600 km to better fit the data. The best-fitting cylindrical anisotropic model for a two-layered IC shows ε_0_ = 1.4%, σ_0_ = −7.3%, γ_0_ = 1.1%, ε_1_ = 1.4%, σ_1_ = −8.7%, and γ_1_ = 1.2% (Fig. 6D), where the subscripts 0 and 1 represent the outer IC and IMIC, respectively. We also plot the fit to relative PKPab-PKIKP travel time residuals in fig. S18.
