# Supplementary material for: Seismic low-velocity equatorial torus in the Earth’s outer core: Evidence from the late–coda correlation wavefield
Source: Sci Adv. 2024 Aug 30;10(35):eadn5562. doi: 10.1126/sciadv.adn5562 (PMC11364092; doi:10.1126/sciadv.adn5562)
Supplement: Supplementary file 1 — Figs. S1 to S19 Tables S1 and S2 [file sciadv.adn5562_sm.pdf]

Science Advances
AAAS

Supplementary Materials for

Seismic low-velocity equatorial torus in the Earth's outer core: Evidence from the late–coda correlation wavefield

Xiaolong Ma and Hrvoje Tkalčić

Corresponding author: Xiaolong Ma, maxiaolong1125@126.com; Hrvoje Tkalčić, hrvoje.tkalcic@anu.edu.au

Sci. Adv. **10**, eadn5562 (2024)
DOI: 10.1126/sciadv.adn5562

This PDF file includes:

Figs. S1 to S19
Tables S1 and S2

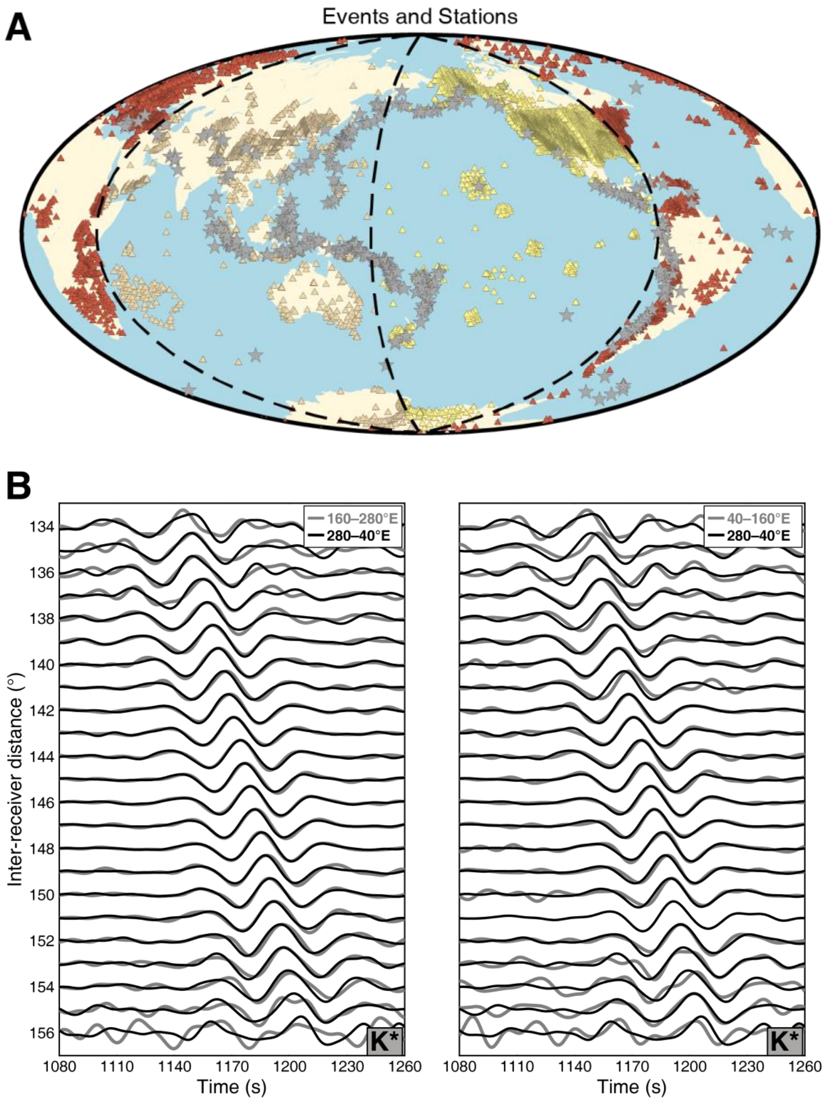

Fig. S1. Waveform comparisons of the observed K* feature between three longitudinally-divided groups. (A) A global distribution of events (grey stars) and stations (triangles) used in this study. The stations are longitudinally divided into three groups (wheat, khaki, and coral triangles). (B) Waveform comparisons of K* between three groups. The longitudinal ranges for these groups are shown in the top right corner.

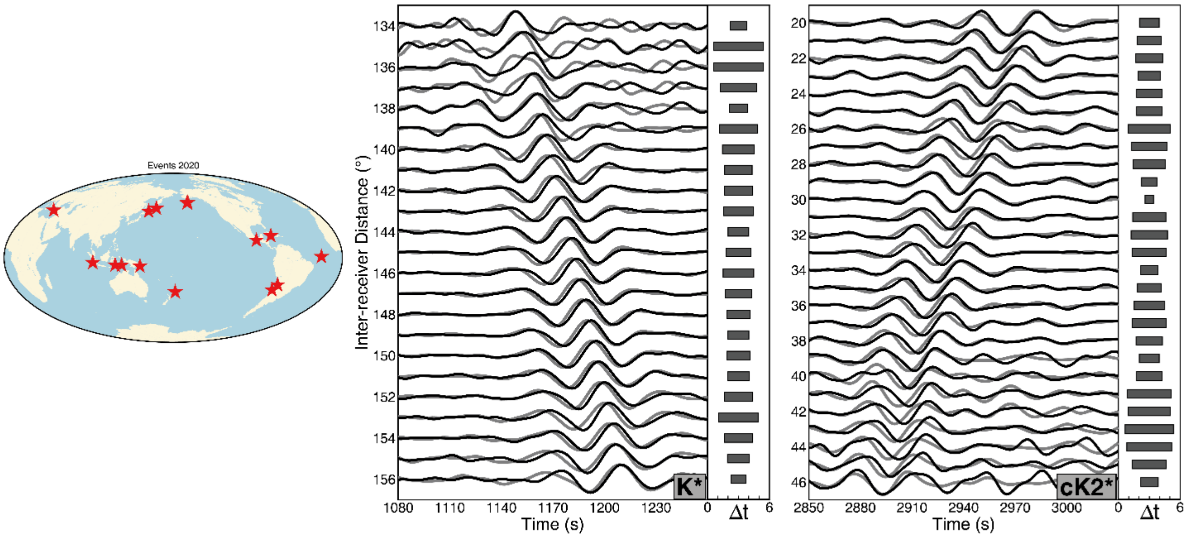

Fig. S2. A subset of earthquakes (red stars) occurred in 2020 used for waveform comparisons of two correlation features (K* and cK2*) in polar (grey) and equatorial (black) groups. The horizontal bars in each side panel indicate the calculated travel-time differences of the features between the polar and equatorial groups.

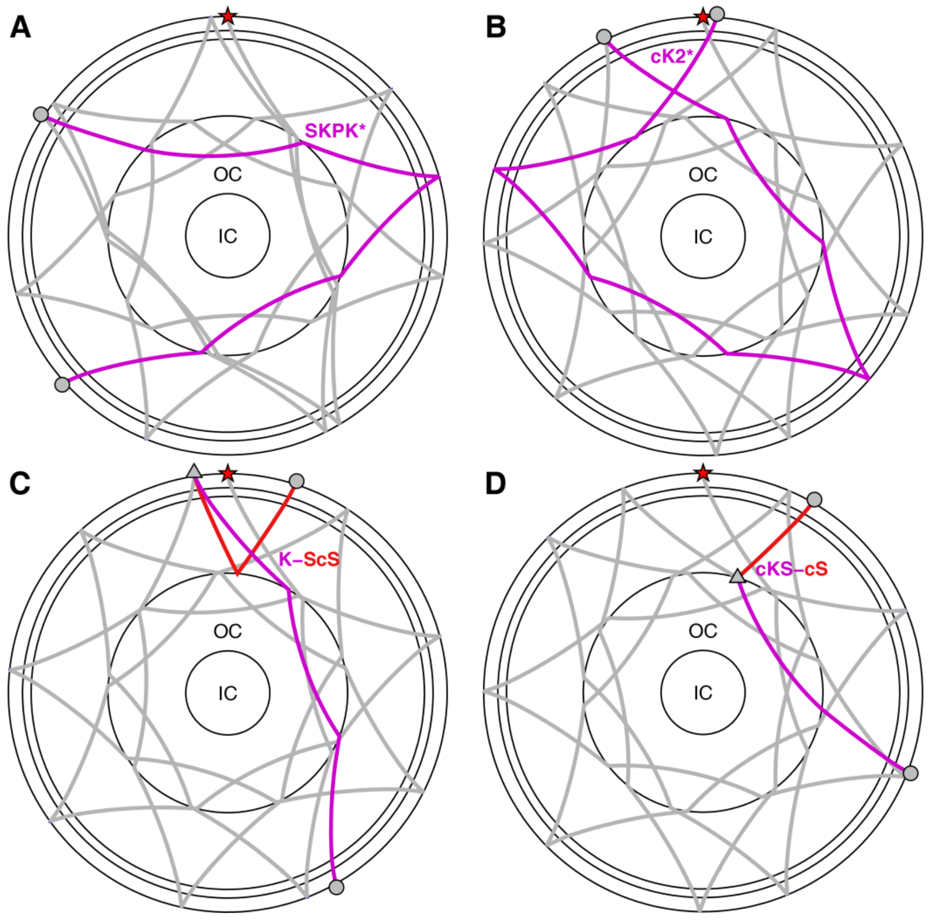

Fig. S3. The ray paths for SKPK* (A), cK2*(B), K-ScS (C), and cKS-cS (D) features. A simple schematic illustration of the generation of these features from one constituent $(PKP)_9X$-$(PKP)_9Y$ is shown here, where X represents SKPPKP, PKPPKPPcP, PKP, and PKS (deep magenta) while Y represents NA, NA, ScS, and PcS (red) correspondingly (NA means there is no phase name). The $(PKP)_9$ (grey) represents PKP traveling up to nine times through the Earth's interior. The grey circles denote the stations, and the red star marks the event. For features with two differential phases (e.g., K-ScS), they are generated by the virtual sources radiating P and S waves that travel along different geometrical ray paths to receivers on the Earth surface with the same ray parameter. The triangles represent such virtual sources either on the surface or CMB.

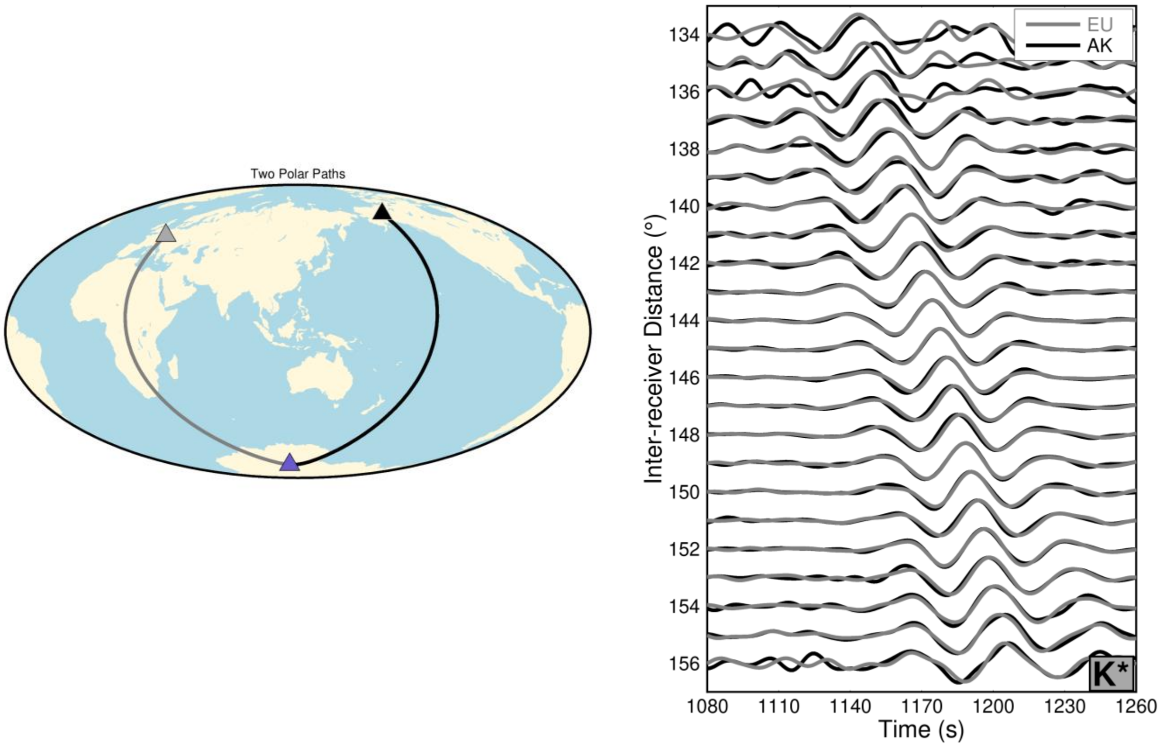

Fig. S4. Waveform comparisons of the observed K* feature for two polar paths. (Left) A schematic illustration of two polar paths connecting the stations in Antarctica with the stations in Europe (EU, grey triangle) and Alaska (AK, black triangle), respectively. (Right) Waveform comparisons of K* between Europe (grey) and Alaska (black) groups.

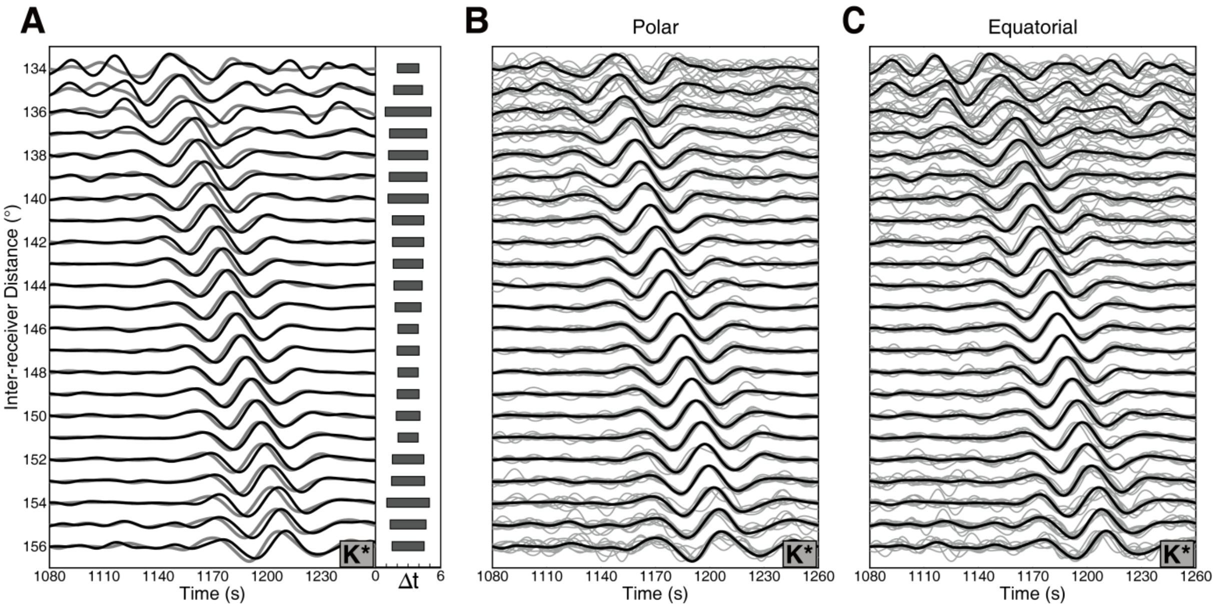

Fig. S5. Waveforms of the observed K* feature from 10 events in polar and equatorial groups. (A) Waveform comparisons of K* between polar (grey) and equatorial (black) groups from 10 events. (B) and (C) show the stacked and individual waveforms of K* for 10 events in the polar and equatorial groups, respectively. Thin grey waveforms are K* calculated for each single event, and the thick black waveforms are linear stacks of K* from 10 events.

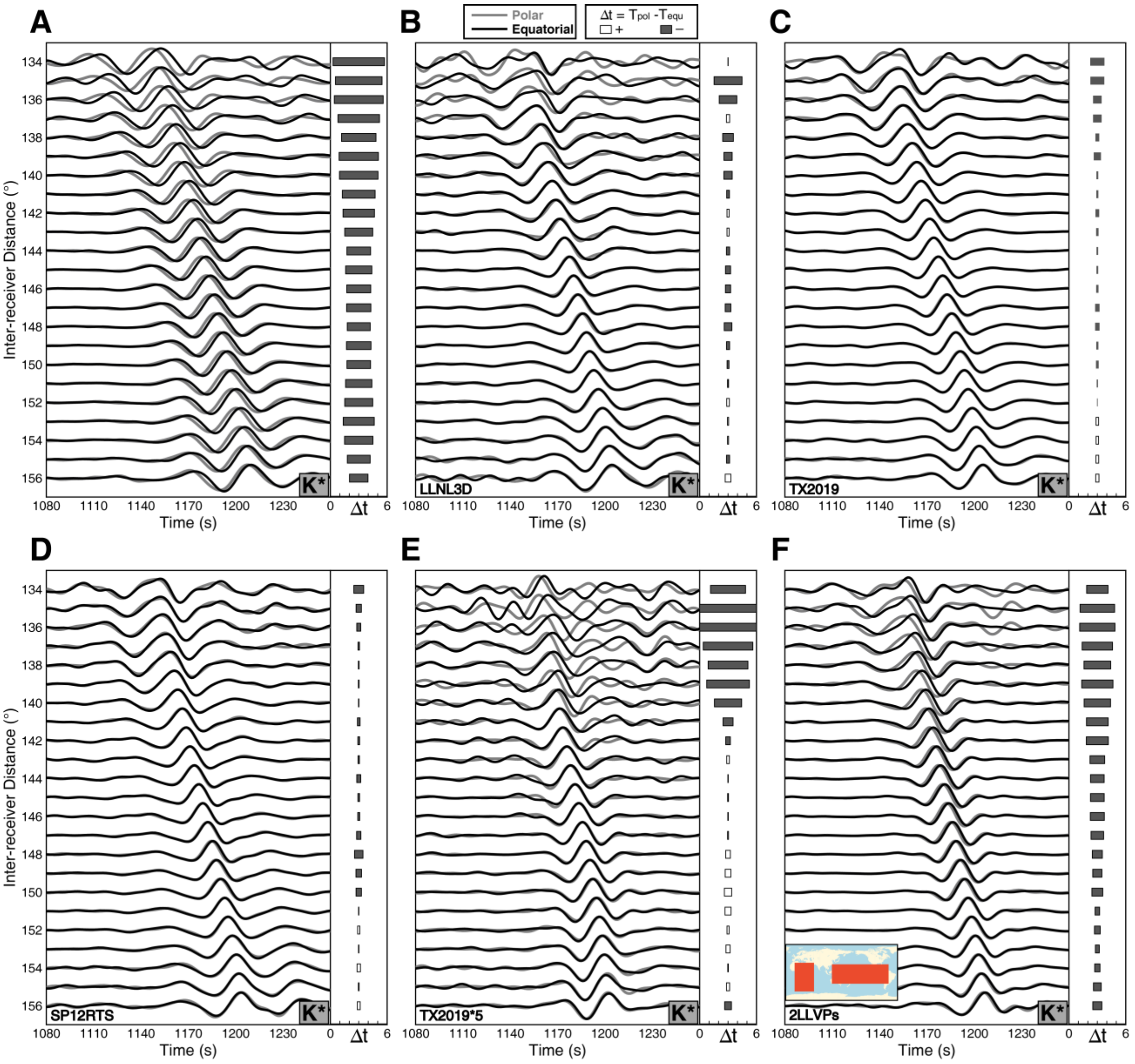

Fig. S6. Waveform comparisons between the polar and equatorial groups for the observed K* and synthesized K* for different models. (A) Observed K* correlation feature. (B) LLNL-G3Dv3 model (*63*). (C) TX2019slab model (*64*). (D) SP12RTS model (*65*). (E) TX2019slab model with the velocity perturbations multiplied by a factor of 5. (F) A two-cuboid model (insert) with P-wave velocity perturbations of -2% and thicknesses of 1500 km (left rectangle) and 500 km (right rectangle), respectively. The two cuboids represent the P-wave velocity equivalents of the two well-documented LLSVPs at the CMB.

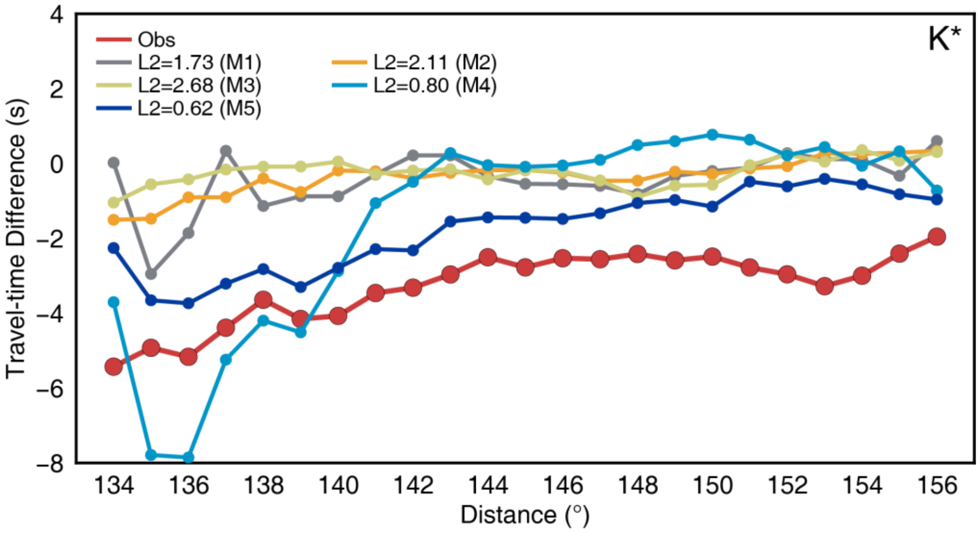

Fig. S7. Plots of the travel-time variations of K* between the polar and equatorial groups for different models compared with the observations. The red line indicates the observed K* travel-time variations. The colored lines represent synthetic K* travel-time differences for individual corresponding models in Fig. S6 (M1: LLNL3D, M2: TX2019, M3: SP12RTS, M4: TX2019*5, M5: 2LLVPs). The L2-norm misfit is calculated for each model.

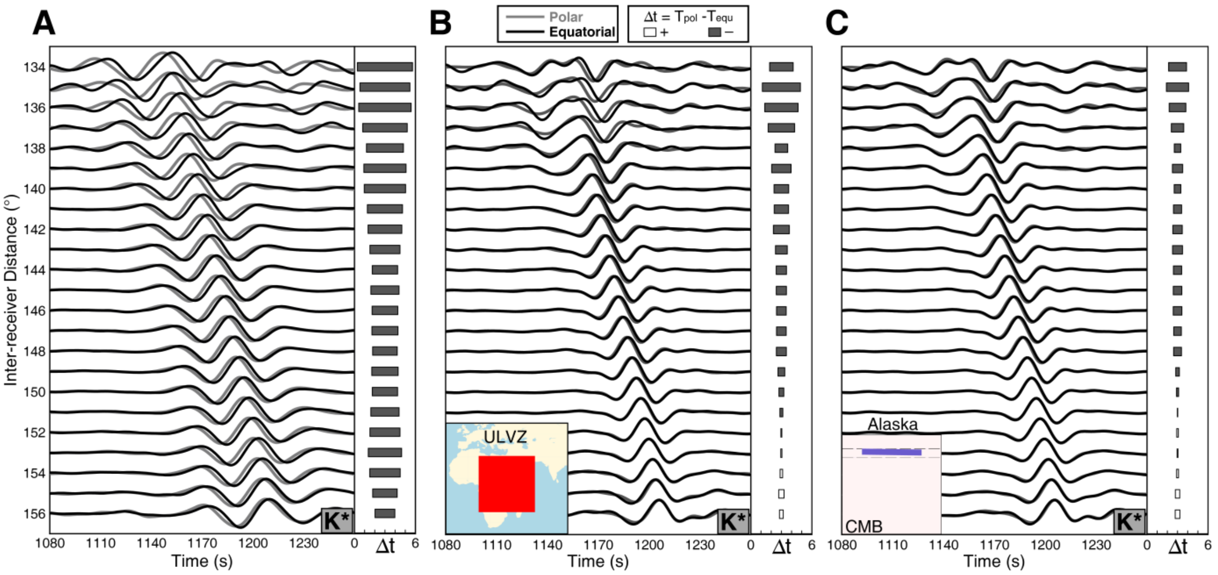

Fig. S8. Waveform comparisons between the polar and equatorial groups for the observed K* and synthesized K* for two regional heterogeneity models. (A) Observed K* correlation feature. (B) A large-scale ULVZ model beneath Africa. (C) A slab model beneath Alaska.

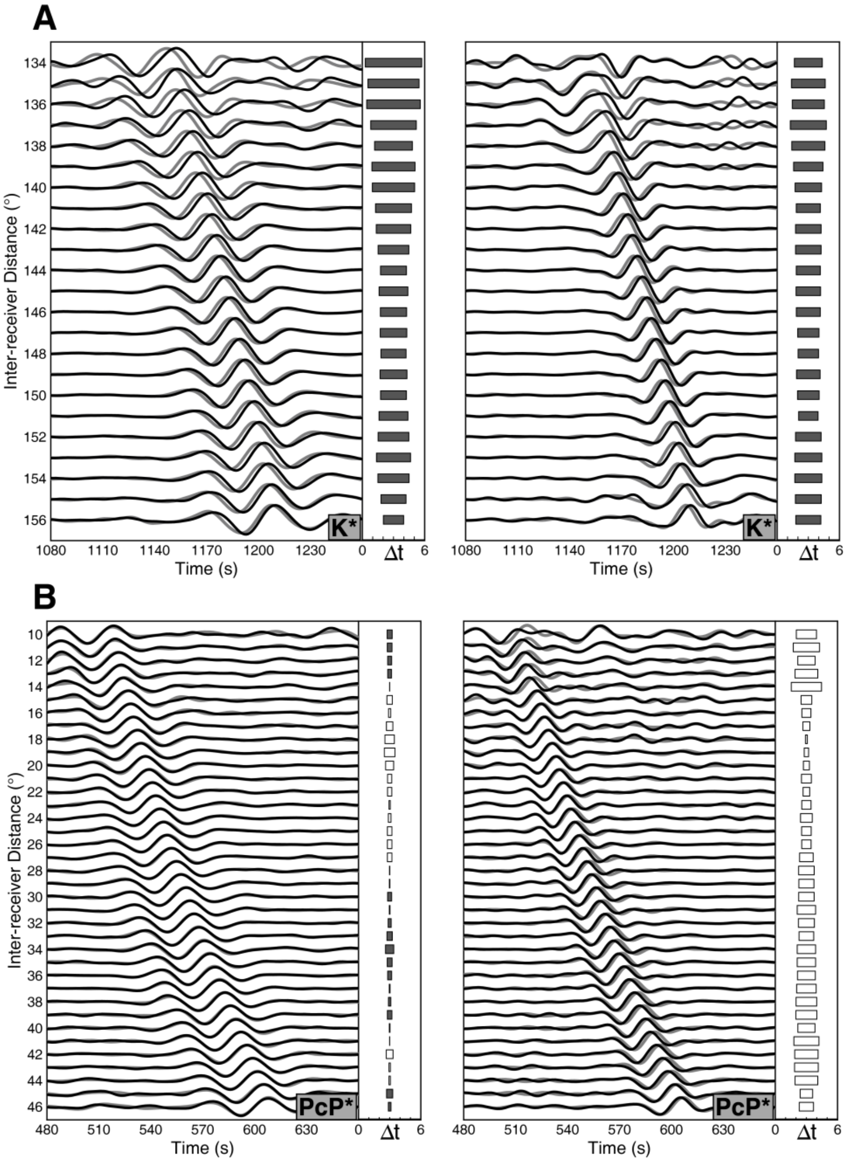

Fig. S9. Observed and synthetic waveform comparisons of K* (A) and PcP* (B) between the polar (grey) and equatorial (black) groups. The observed waveforms of K* and PcP* are shown in the left. The synthetic waveforms for the model with the CMB topography increased by 20 km within the latitudes of ±30 ° are in the right column.

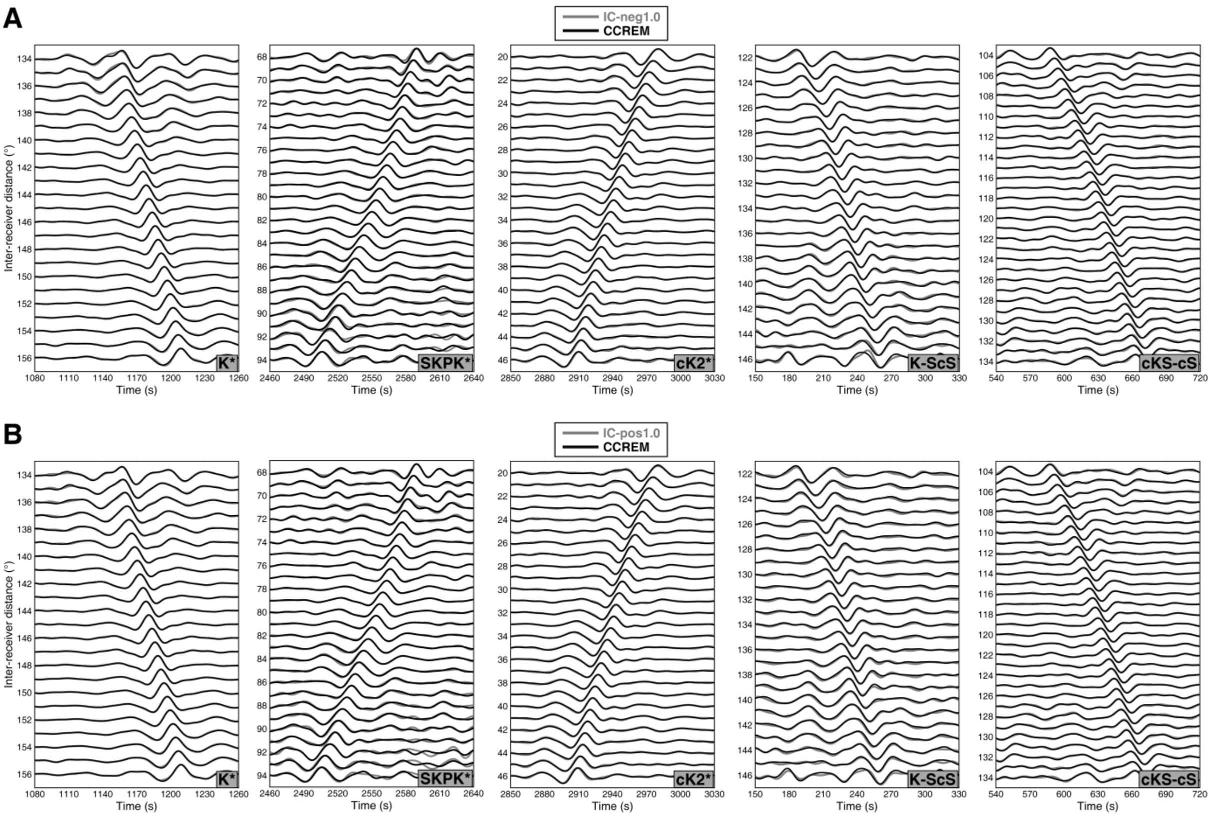

Fig. S10. Synthetic waveform comparisons calculated based on the CCREM (black) model and two CCREM-perturbed models (grey) for the correlation features K*, SKPK*, cK2*, K-ScS and cKS-cS. In the two CCREM-perturbed models, the P-wave velocity in the IC is varied by -1.0% (A) and +1.0% (B), respectively. IC-neg1.0 and IC-pos1.0 mean the IC P-wave velocity is perturbed by -1.0% and +1.0%, respectively.

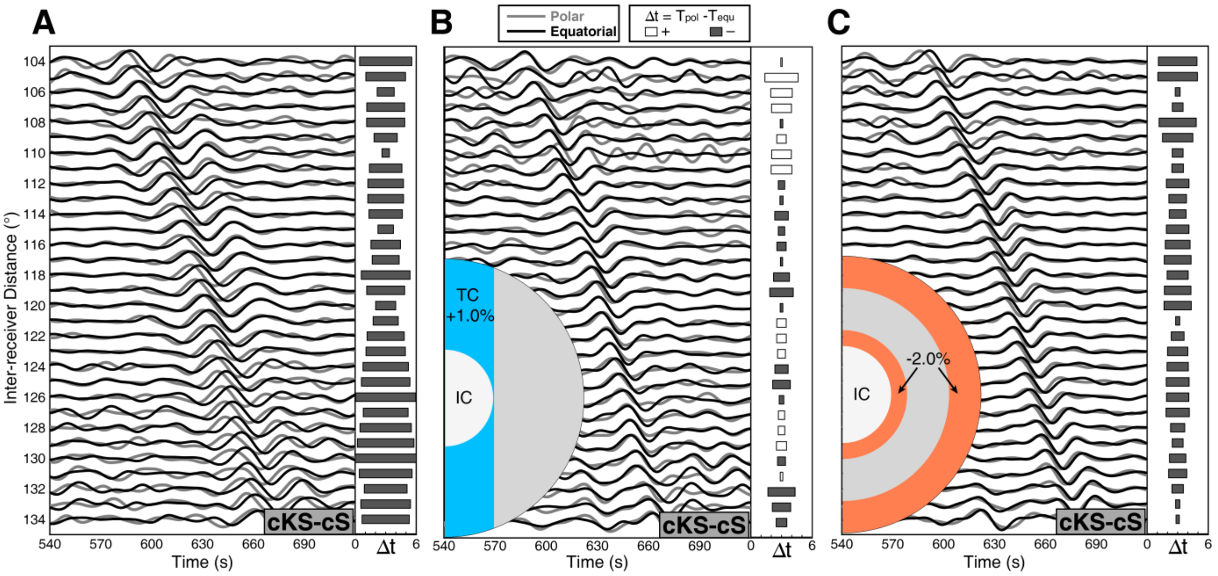

Fig. S11. The observed and synthetic waveform comparisons of the cKS-cS feature between the polar (grey) and equatorial (black) groups. The observed cKS-cS is shown in (A) for comparison. The synthetic waveforms for the TC and the layered OC models are shown in (B) and (C).

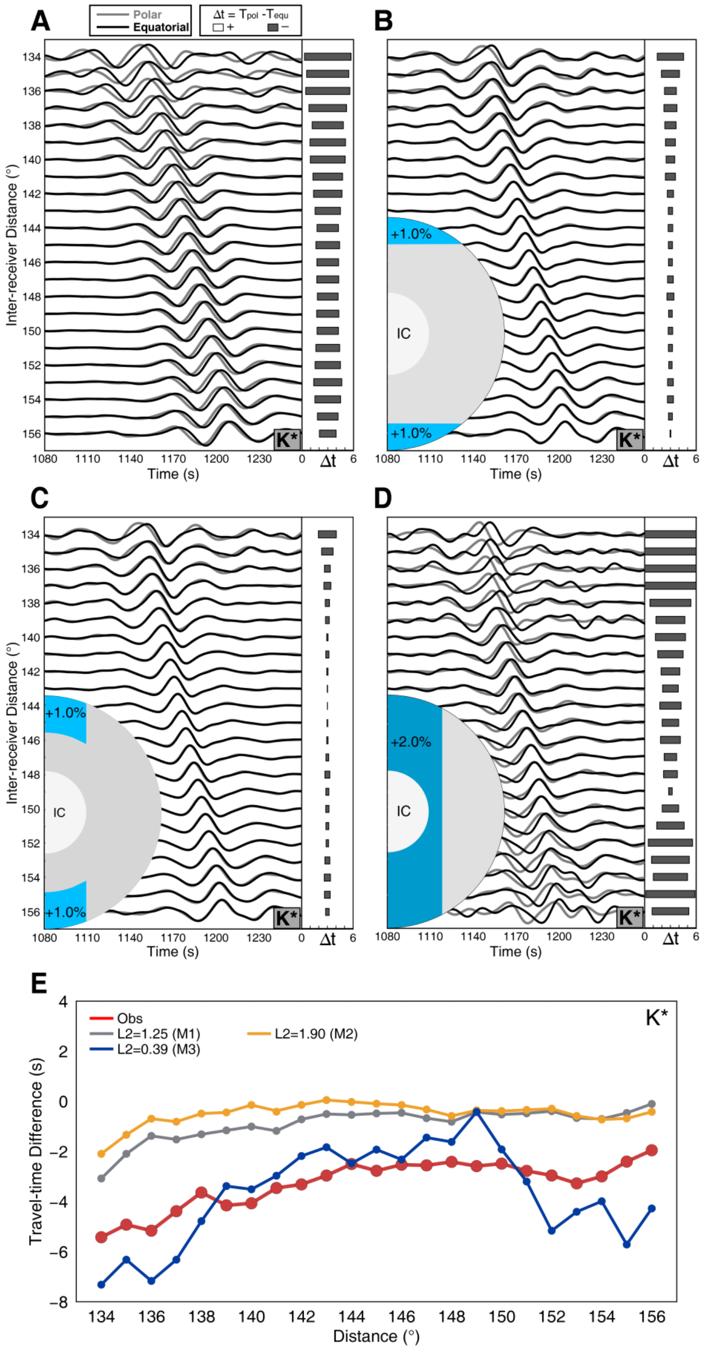

Fig. S12. Waveform comparisons between the polar and equatorial groups for the observed K* and synthesized K* using different models (M1–M3). (A) Observed K* correlation feature. (B) A polar-cap model showing +1% velocity perturbation with the largest thickness of 800 km (light blue). (C) A heterogeneous model showing +1% velocity perturbation with a thickness of about 1050 km (light blue). (D) A columnar heterogeneity with +2% velocity perturbation and a cylinder radius of 1621 km (slate blue). (E) Plots of the travel-time differences for the K* feature between the polar and equatorial groups for models in (B-D) relative to the observations (M1, M2 and M3 indicate the models in B-D, respectively).

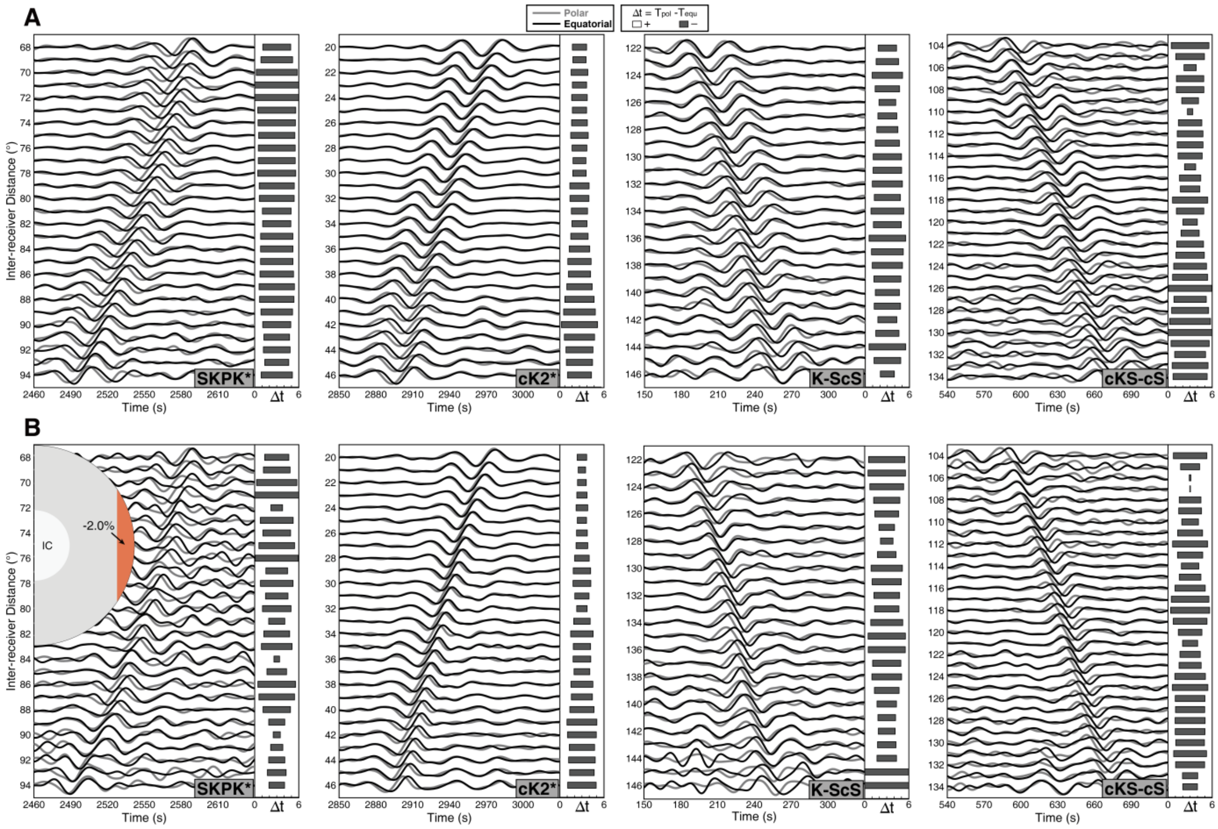

Fig. S13. Observed and synthetic waveform comparisons of four correlation features between the polar and equatorial groups. (A) Observed waveform comparisons of the selected correlation features (SKPK*, cK2*, K-ScS, and cKS-cS) between the polar and equatorial groups. (B) Waveform comparisons of corresponding synthetic features through the preferred OC model using PREM as a background model (top left corner in B) in the simulation.

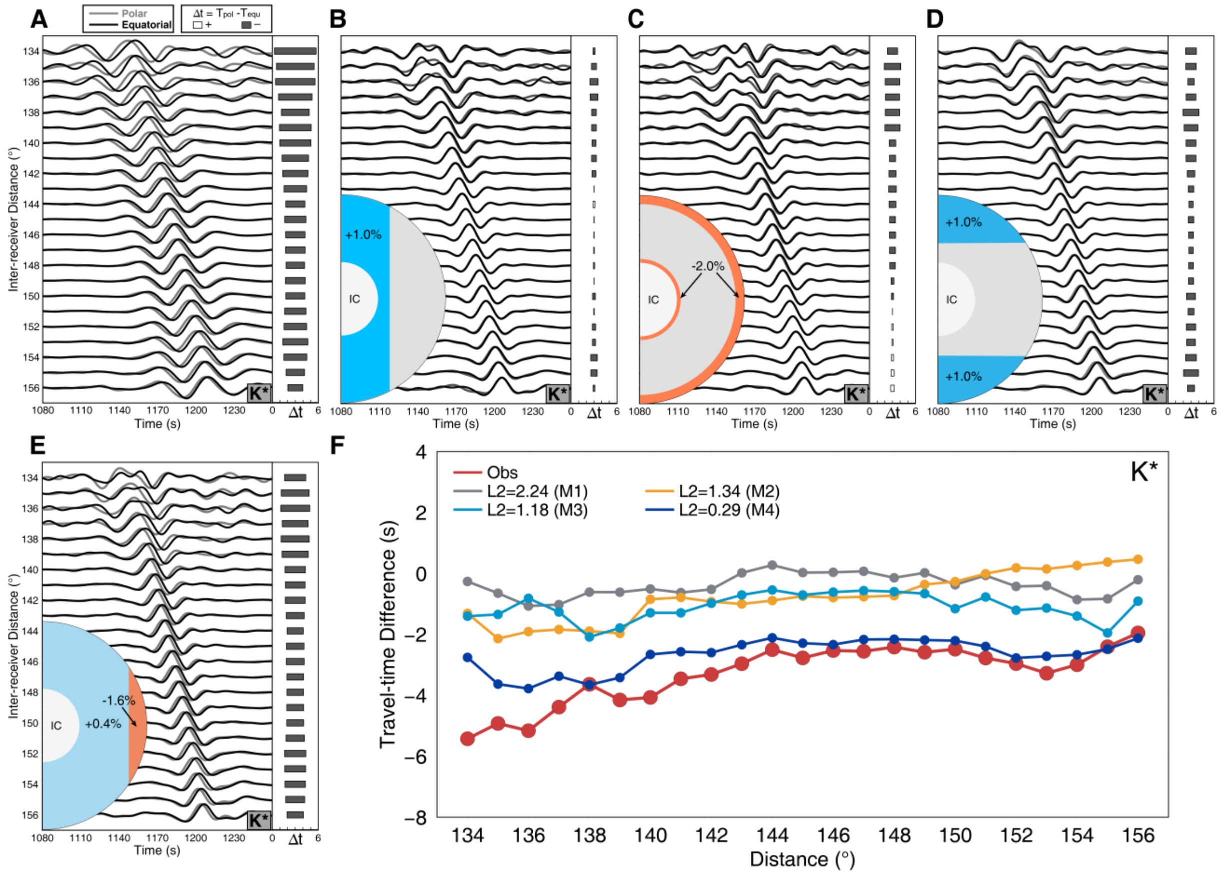

Fig. S14. Waveform comparisons between the polar and equatorial groups for the observed K* and synthesized K* through some variants of the preferred OC models. (A) Observed K* correlation feature. (B) A tangent cylinder model showing +1% velocity perturbation with a radius of about 1617 km. (C) A layered model with a low seismic velocity of -2% at the top and bottom of the OC. The thicknesses for the low-velocity regions are 300 km and 150 km at the top and bottom, respectively. (D) A polar-cap model showing +1% velocity perturbation with the largest thickness of 1600 km. (E) A model with a low seismic velocity of -1.6% in the equatorial torus and a slightly high velocity of +0.4% in the remaining part. (F) Plots of the travel-time differences for the K* feature between the polar and equatorial groups for models in (B-E) relative to the observations (M1, M2, M3 and M4 indicate the models in B-E, respectively).

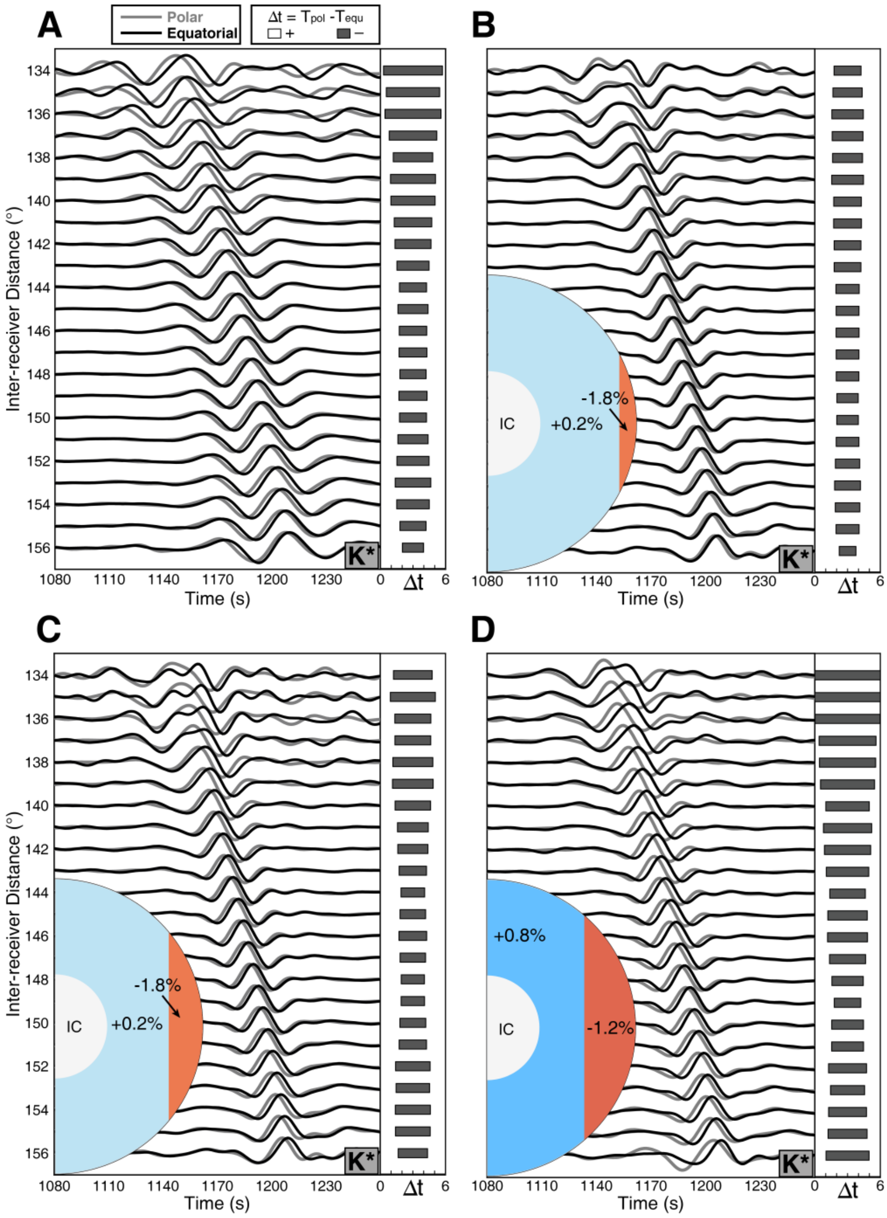

Fig. S15. Waveform comparisons between the polar and equatorial groups for the observed K* and synthesized K* through some variants of the preferred OC models. In these models, we add a 150-km-thick buffer zone. (A) Observed K* correlation feature. (B) A model showing -1.8% velocity perturbation in the equatorial torus with the thickest part reaching 400 km beneath the CMB and +0.2% in the rest of OC. (C) Similar to (B), a model with the thickest part reaching 800 km beneath the CMB. (D) A model showing -1.2% velocity perturbation in the equatorial torus with the thickest part reaching 1200 km beneath the CMB and +0.8% in the rest of OC.

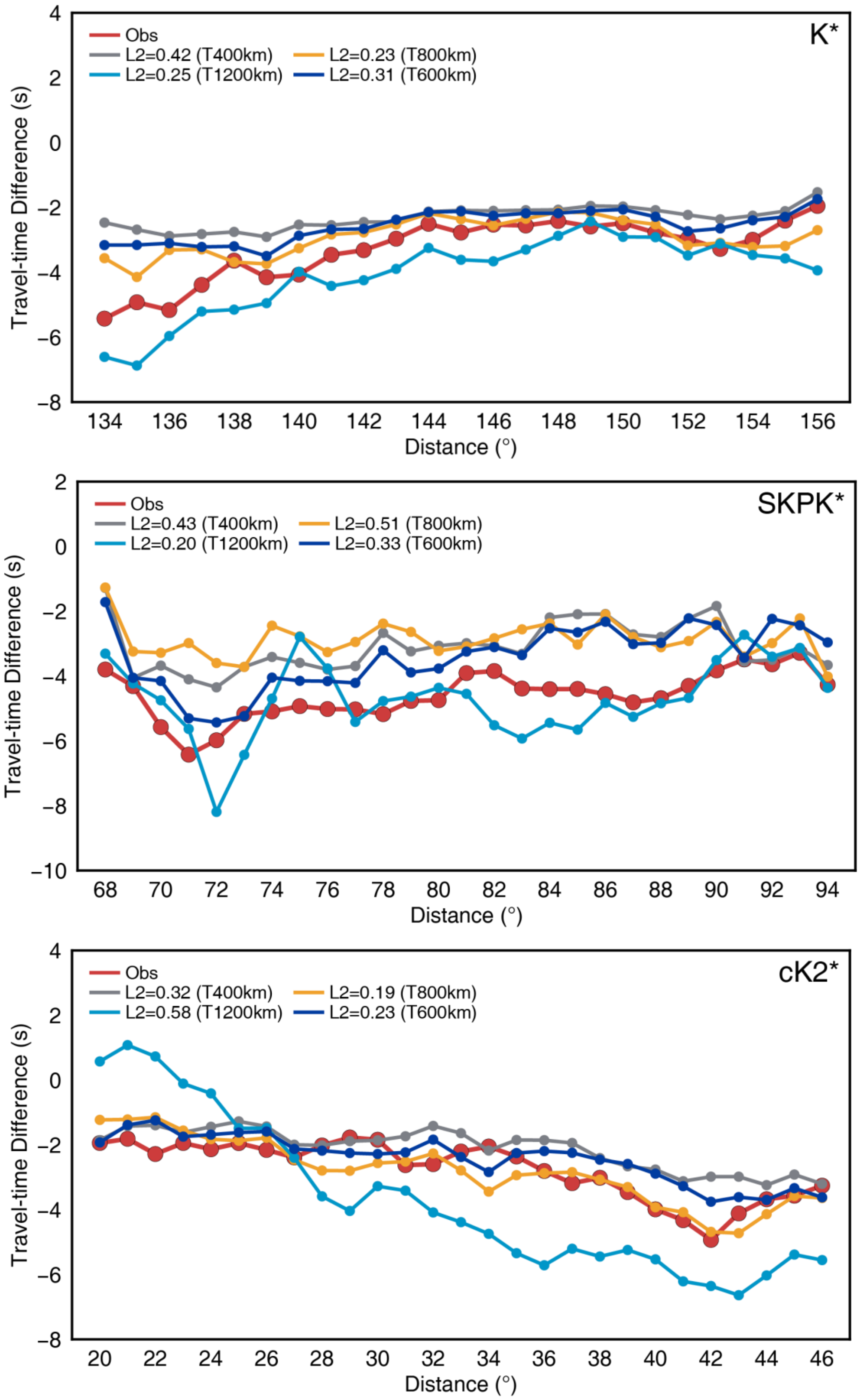
K*
Obs
L2=0.42 (T400km)
L2=0.23 (T800km)
L2=0.25 (T1200km)
L2=0.31 (T600km)
Travel-time Difference (s)
Distance (°)
SKPK*
Obs
L2=0.43 (T400km)
L2=0.51 (T800km)
L2=0.20 (T1200km)
L2=0.33 (T600km)
Travel-time Difference (s)
Distance (°)
cK2*
Obs
L2=0.32 (T400km)
L2=0.19 (T800km)
L2=0.58 (T1200km)
L2=0.23 (T600km)
Travel-time Difference (s)
Distance (°)

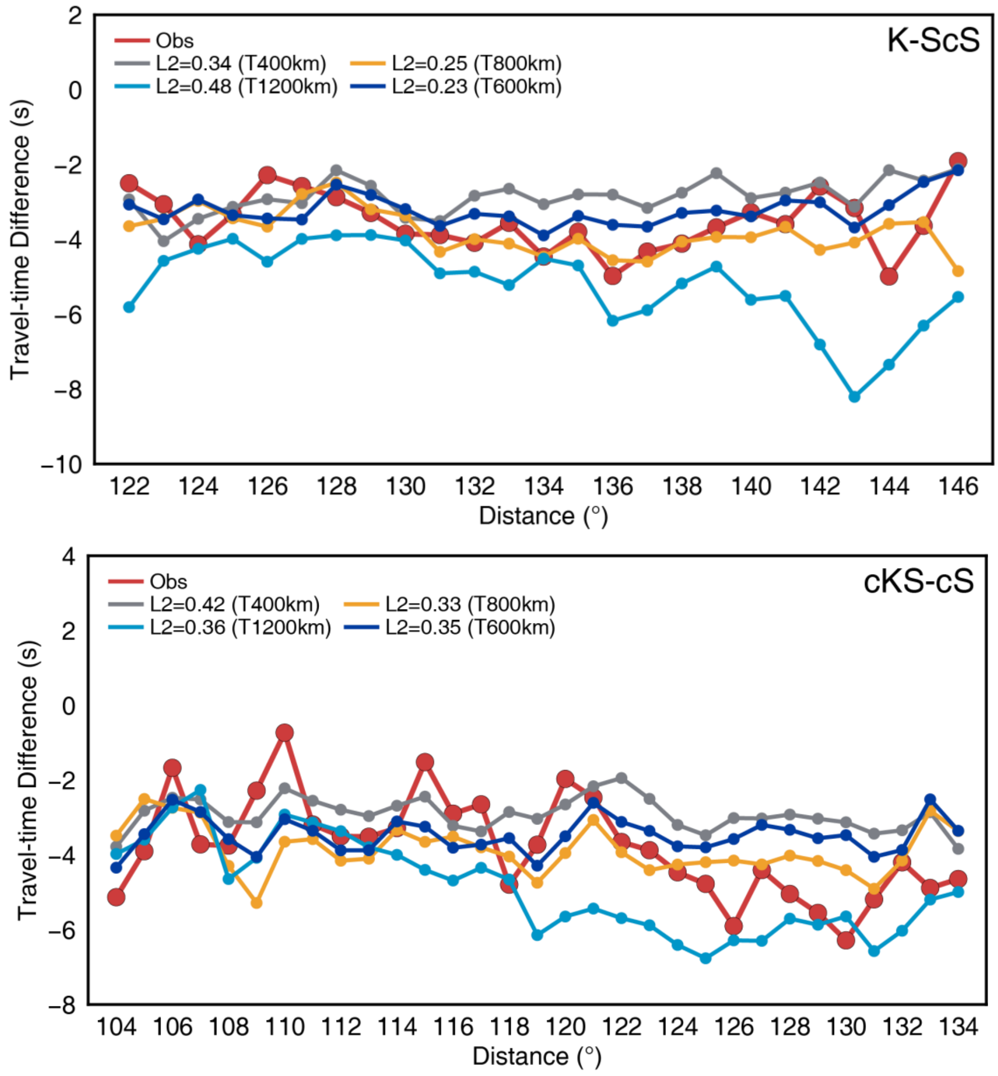

Fig. S16. Plots of the travel-time variations for all selected correlation features between the polar and equatorial groups for models in Fig. S15 compared with the observations. T400km, T800km and T1200km represent the models shown in Fig. S15, respectively. T600km represents the model in Fig. 5.

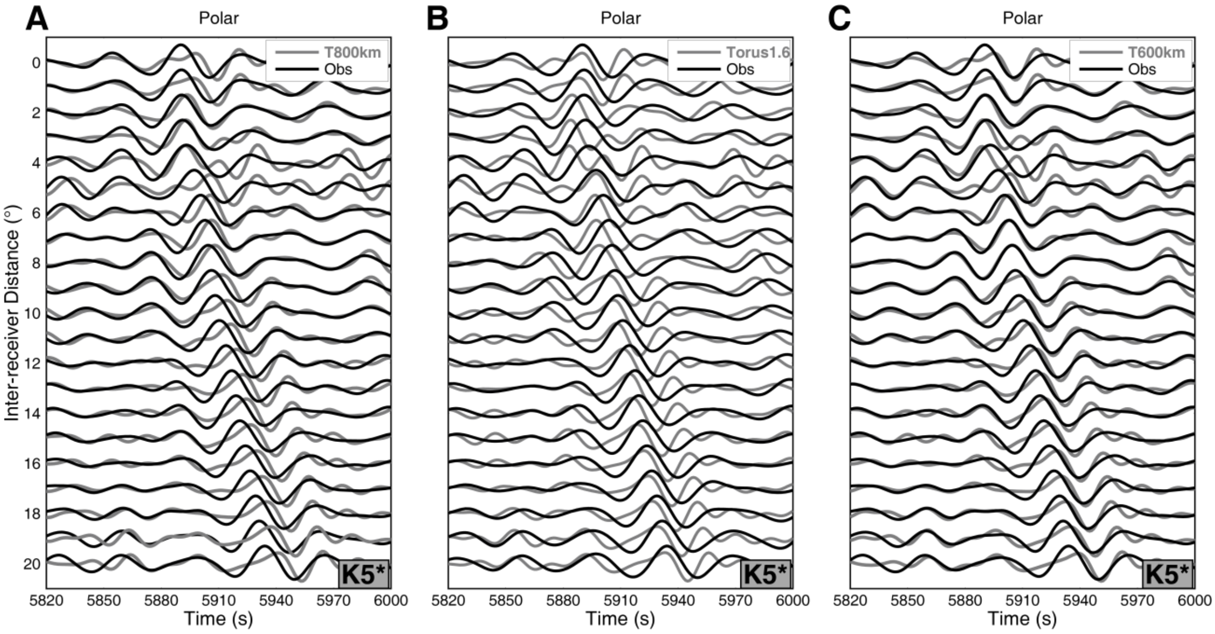

Fig. S17. Waveform comparisons of synthesized (grey) and observed (black) K5* in the polar group through three torus models. T800km and T600km indicate the torus models showing -1.8% velocity perturbation in the equatorial torus with the thickest part reaching 800 km and 600 km beneath the CMB, respectively. Torus1.6 represents the model dispalying a low seismic velocity of -1.6% in the equatorial torus with the thickest part reaching 600 km and a weakly-elevated velocity of +0.4% in the remaining part.

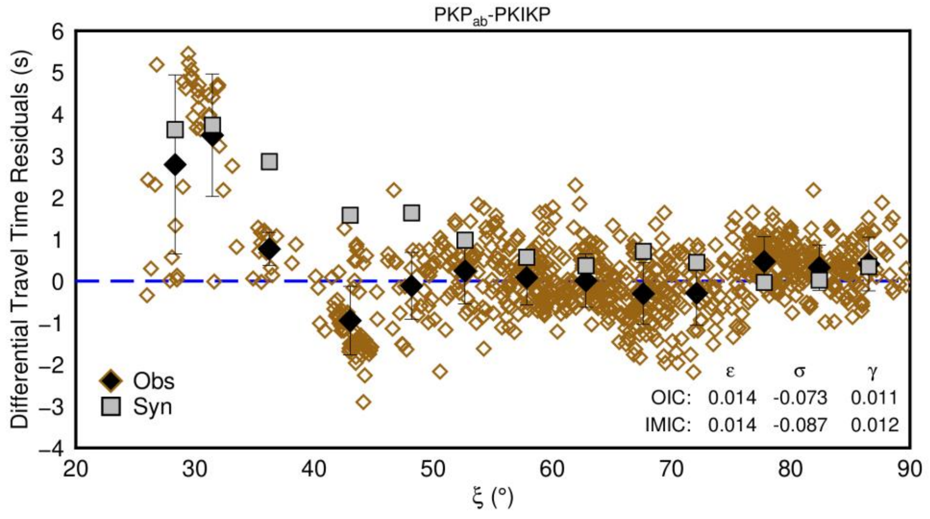

Fig. S18. Comparison of the observed and predicted PKPab-PKIKP differential travel time residuals as a function of the angle between the PKIKP ray path in the IC and rotation axis. The brown diamonds show the observed travel time residuals and the black diamonds indicate the binned data with 1-σ error bars calculated for every 5°. The grey squares denote the binned synthetic PKPab-PKIKP travel time residuals calculated for the TX2019slab + OC torus + IC anisotropic model. The anisotropic parameters of the IC model are shown in the bottom right corner.

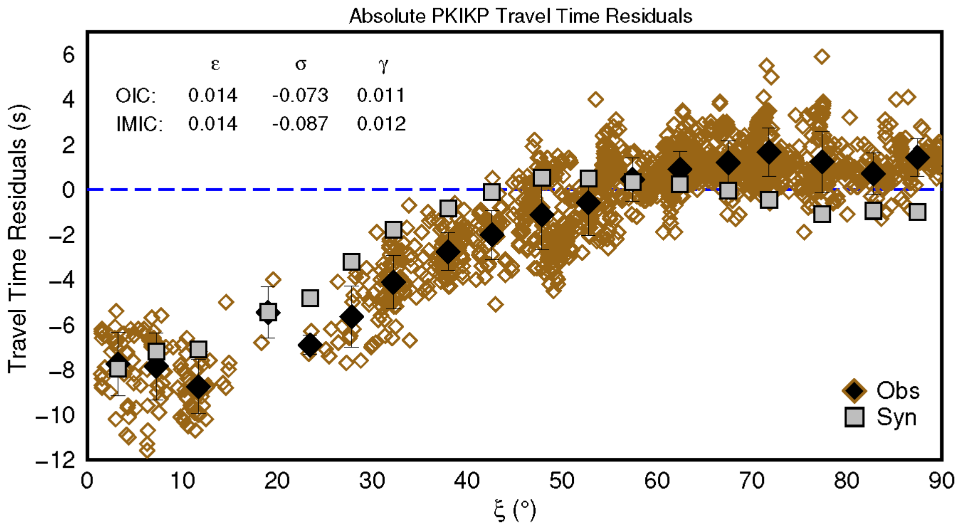

Fig. S19. Similar to Fig. 6D, observed and predicted absolute PKIKP travel time anomalies for the model without the OC torus structure. Black diamonds are binned data with 1-σ error bars and a bin size of 5°. The grey squares indicate the synthetic binned data calculated for the model, including the mantle heterogeneity and IC anisotropic structure shown on the top left.

Table S1. L2-norm misfit values for the K* correlation feature for the models tested in this study.

Model Name	L2-norm misfit for K*
PREM	1.46
LLNL3D	1.73
TX2019	2.11
SP12RTS	2.68
TX2019*5	0.80
2LLVPs	0.62
ULVZ	0.98
AK-Slab	1.51
Tangent Cylinder	1.85
Layered OC-1	2.01
PREM-TORUS-Neg2.0	0.28
Polar-Cap1	1.25
Polar-Cap2	1.90
Polar-Cap3	1.18
TC-Col-Pos2	0.39
TC-Col-Pos1	2.24
Layered OC-2	1.34
TORUS-600km-Neg1.6	0.29
TORUS-400km-Neg1.8	0.42
TORUS-800km-Neg1.8	0.23
TORUS-1200km-Neg1.2	0.25
TORUS-600km-Neg1.8	0.31

Table S2. L2-norm misfit values for other correlation features for various torus models.

Model Name	K*	SKPK*	cK2*	K-ScS	cKS-cS	Total
PREM-TORUS-Neg2.0	0.28	0.41	0.24	0.41	0.39	1.73
TORUS-600km-Neg1.6	0.29	0.47	0.20	0.18	0.37	1.51
TORUS-400km-Neg1.8	0.42	0.43	0.32	0.34	0.42	1.93
TORUS-800km-Neg1.8	0.23	0.51	0.19	0.25	0.33	1.51
TORUS-1200km-Neg1.2	0.25	0.20	0.58	0.48	0.36	1.87
TORUS-600km-Neg1.8	0.31	0.33	0.23	0.23	0.35	1.45
